# Supplementary material for: Biomolecular condensates can both accelerate and suppress aggregation of α-synuclein
Source: Sci Adv. 2022 Dec 2;8(48):eabq6495. doi: 10.1126/sciadv.abq6495 (PMC10942789; doi:10.1126/sciadv.abq6495)
Supplement: Supplementary file 1 — Supplementary Methods Figs. S1 to S13 Supplementary Model References [file sciadv.abq6495_sm.pdf]

Science Advances
AAAS

Supplementary Materials for

Biomolecular condensates can both accelerate and suppress aggregation of α-synuclein

Wojciech P. Lipiński *et al.*

Corresponding author: Evan Spruijt, e.spruijt@science.ru.nl

Sci. Adv. **8**, eabq6495 (2022)
DOI: 10.1126/sciadv.abq6495

This PDF file includes:

Supplementary Methods
Figs. S1 to S13
Supplementary Model
References

Supplementary Methods

Reagents (supplementary)

Insulin (human, recombinant) was purchased from FujiFilm/Wako Pure Chemical Corporation. 5(6)-Carboxyfluorescein N-hydroxysuccinimide ester (FAM-NHS) and sodium bicarbonate were purchased from Sigma-Aldrich. Other chemicals used in the supplementary methods were mentioned in the main text.

ThT aggregation kinetics under confocal microscope

ThT aggregation kinetics under microscope experiment was performed using Leica SP8x confocal microscope equipped with 40x magnification water-immersion objective. Samples were placed in 18-well chambered glass coverslips (Ibidi GmbH, Germany), previously modified with PLL-g-PEG and the whole setup was incubated at 37 °C during the experiment. Composition of samples was the same as for the plate-reader ThT aggregation kinetic assays. Samples were excited at 405 nm and the emission was recorded at 440-600 nm. Fluorescence intensity images were saved in 8-bit 512x512 pixels format.

Determination of critical salt concentration of coacervate systems with αSyn variants

Critical salt concentration was determined by titration in a plate reader. Samples of 50 µl with the same composition as for the ThT aggregation kinetic assay but without NaCl (50 mM HEPES, 100 µM EDTA, 20 µM ThT, and 40 µM FL-αSyn or αSyn-108, or 160 µM of NACore) were placed in wells of 384-well plate (non-binding, black walls, Greiner Bio-One GmbH, Austria) and titrated by adding stepwise 750 mM NaCl solution. Each step consisted of adding 2 µl of NaCl solution to each well, waiting 20 seconds and measuring absorbance at 600 nm. Experiment was repeated 3 times for each composition. Critical salt concentration was determined by reading x-coordinate of the intersection of the tangent of the absorbance vs. NaCl concentration curve at its maximal slope and the baseline.

Labelling of insulin

Insulin was labelled with FAM-NHS using the following method. Insulin was dissolved at 5 mg/ml concentration in sodium bicarbonate solution (0.1 M). FAM-NHS was dissolved in DMF at 10 mg/ml. Solution of FAM-NHS (54 µl) was added to solution of insulin (1.32 ml) and the mixture was stirred gently at 4 °C overnight. Subsequently, insulin was separated from unbound dye using Amicon Ultra-15 centrifugal filters with 3 kDa MWCO, by washing with 0.1 M carbonate buffer (4 times) 0.005 M carbonate buffer (5 times).

Partitioning of FAM-labelled insulin

Partitioning of FAM-labelled insulin was studied the same way as described for labelled protein in the main text.

ThT aggregation kinetics assays (insulin)

Aggregation assays were performed analogously to assays described in the main text. The same buffer composition was used (50 mM HEPES, 100 mM NaCl, 100 µM EDTA, 20 µM ThT). Insulin was first dissolved in 10 mM hydrochloric acid and this stock was further diluted to obtain 50 µM insulin concentration in the aggregation assays. Other conditions remained as described in the main text. Kinetic parameters were extracted as described in the main text.

Preparation of samples and transmission electron microscopy (insulin)

Samples of insulin aggregates were prepared using samples from 384-well plate after the aggregation assay. Content of selected wells that shown aggregation in the ThT assay were mixed with a pipette and transferred onto a TEM grid (EM-Tec formvar carbon support film on copper, 300 square mesh, Micro to Nano, the Netherlands). Samples were blotted with filter paper, stained with 1.5 µl of 2% (w/w) sodium phosphotungstate solution (adjusted to pH 7.4), washed with 2 µl of water left to dry overnight. Imagining was performed using JEOL JEM-1400 FLASH.

Statistical analysis (supplementary)

Microscopy images were analysed using FIJI distribution of ImageJ. Violin plots were prepared according to the description under fig. S4 and S9.

Supplementary Figures

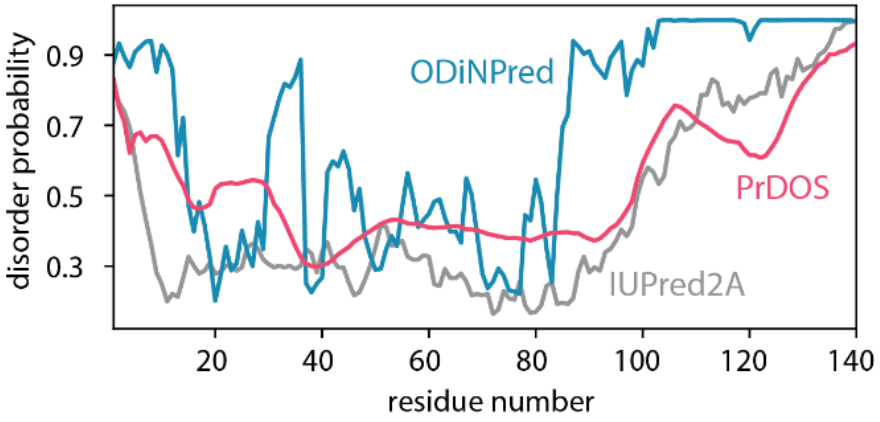

Fig. S1. Comparison of predicted disorder probability for FL-αSyn using different predictors. Predictors used are indicated by the labels: PrDOS (75), ODiNPred (76), and IUPred2A (77).

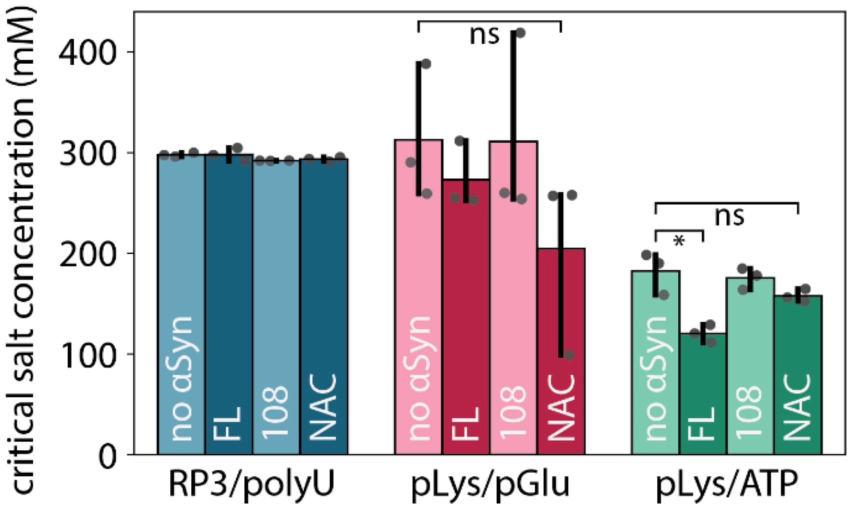

Fig. S2. Critical salt concentration of coacervate systems without and with αSyn variants. All coacervate systems were tested in with FL-αSyn, αSyn-108 and NACore**.** Differences between selected samples were tested for statistical significance (student's t-test) in coacervate droplets-supernatant control pairs. "ns" indicates values above 0.05, single asterisk indicates α<0.05.

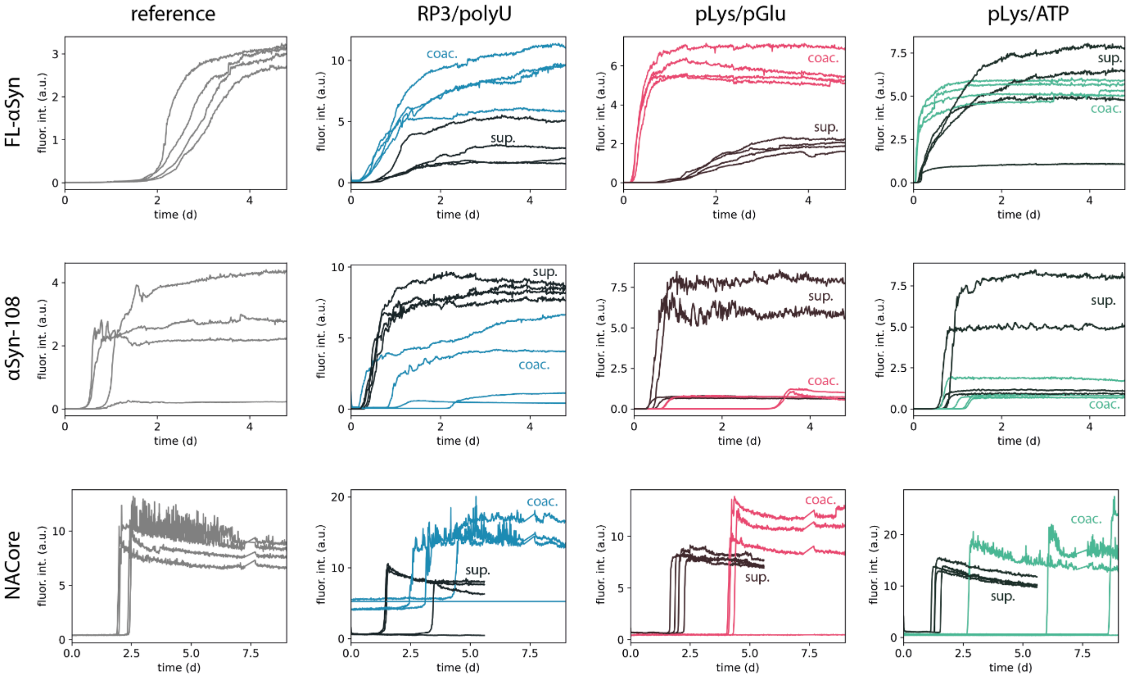

Fig. S3. The kinetics of aggregation of different αSyn variants is altered by coacervates. Lines correspond to single aggregation experiments (ThT fluorescence intensity) of different αSyn variants in buffer (reference, grey traces), in the presence of coacervates (coloured traces), or in the presence of coacervate supernatants (dark traces).

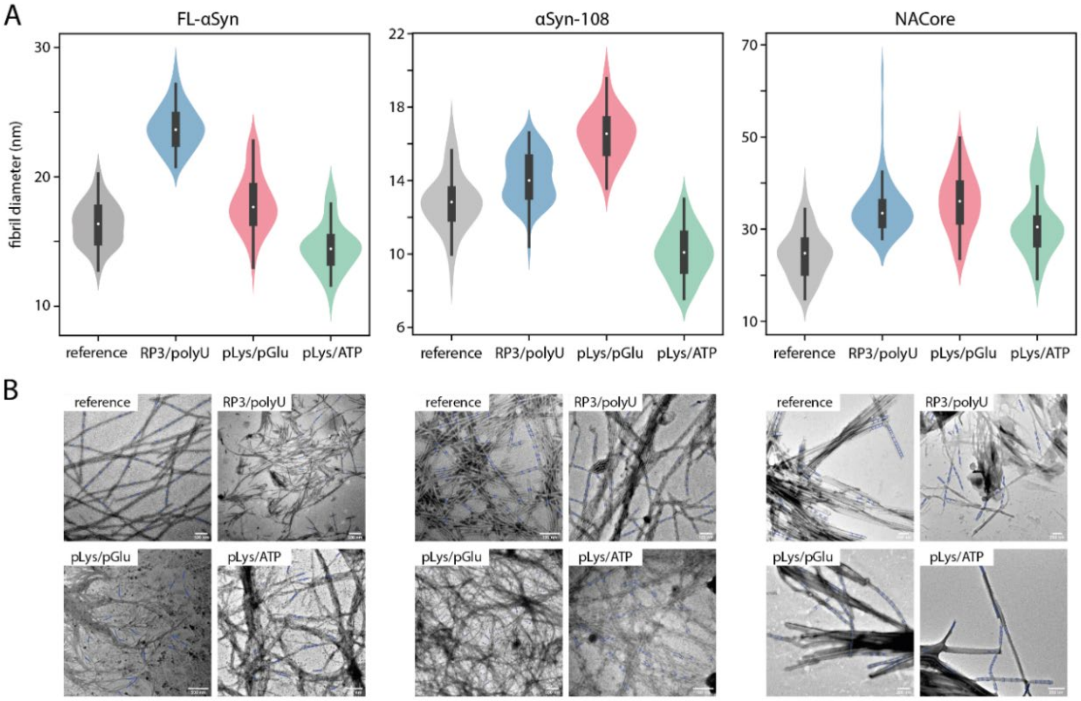

Fig. S4. Characteristics of αSyn fibrils aggregated in absence and presence of coacervates. (**A**) Distribution of fibril thickness formed by different αSyn variants in the absence (blank) or presence of coacervate systems (*n*=50). Violin plots were prepared using Gaussian kernels with bandwidth determined automatically using Scott's method. (**B**) TEM images of the fibrils formed by different αSyn variants in the absence (blank) or presence of coacervate systems. Blue marks indicate places where the diameter was measured.

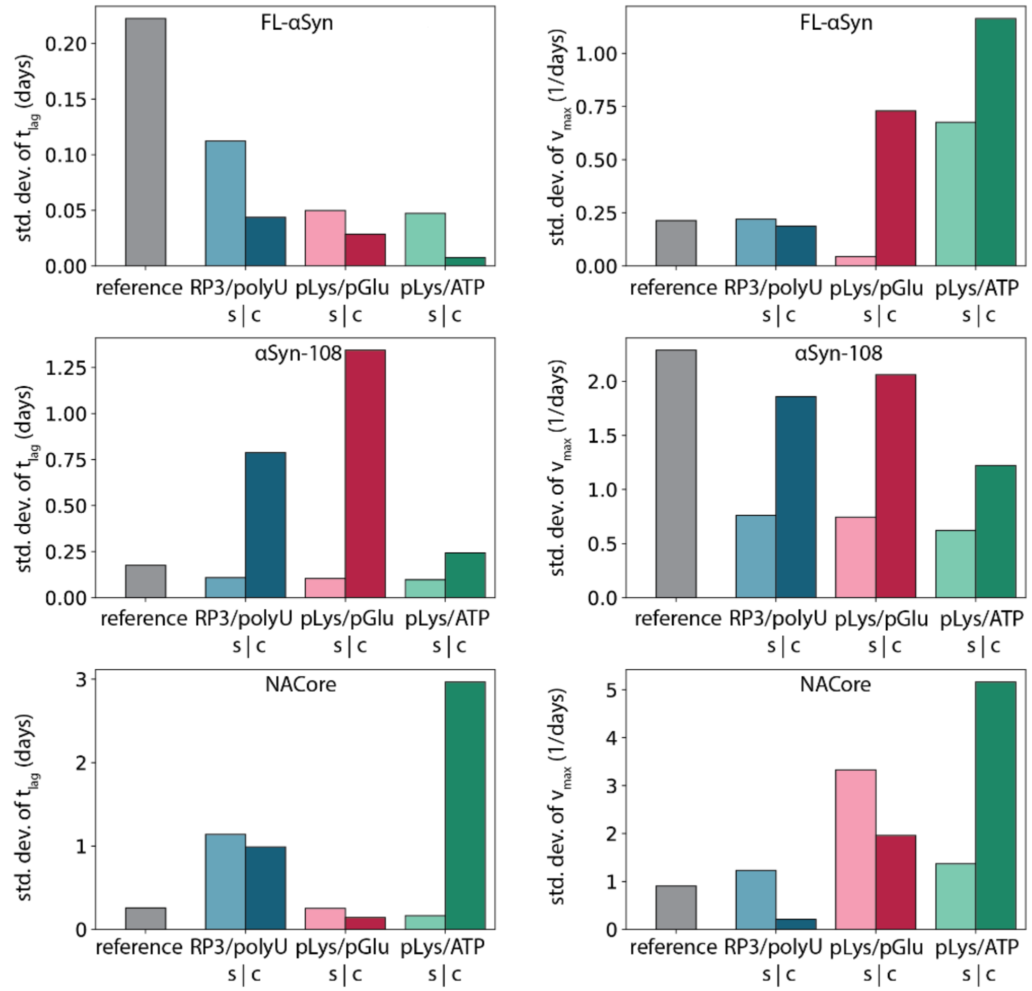

Fig. S5. Variability in lag time and maximum aggregation rate is altered by coacervates. Standard deviation of aggregation parameters for all protein variants and all coacervate systems (supernatant – s, coacervate – c) and for the reference sample.

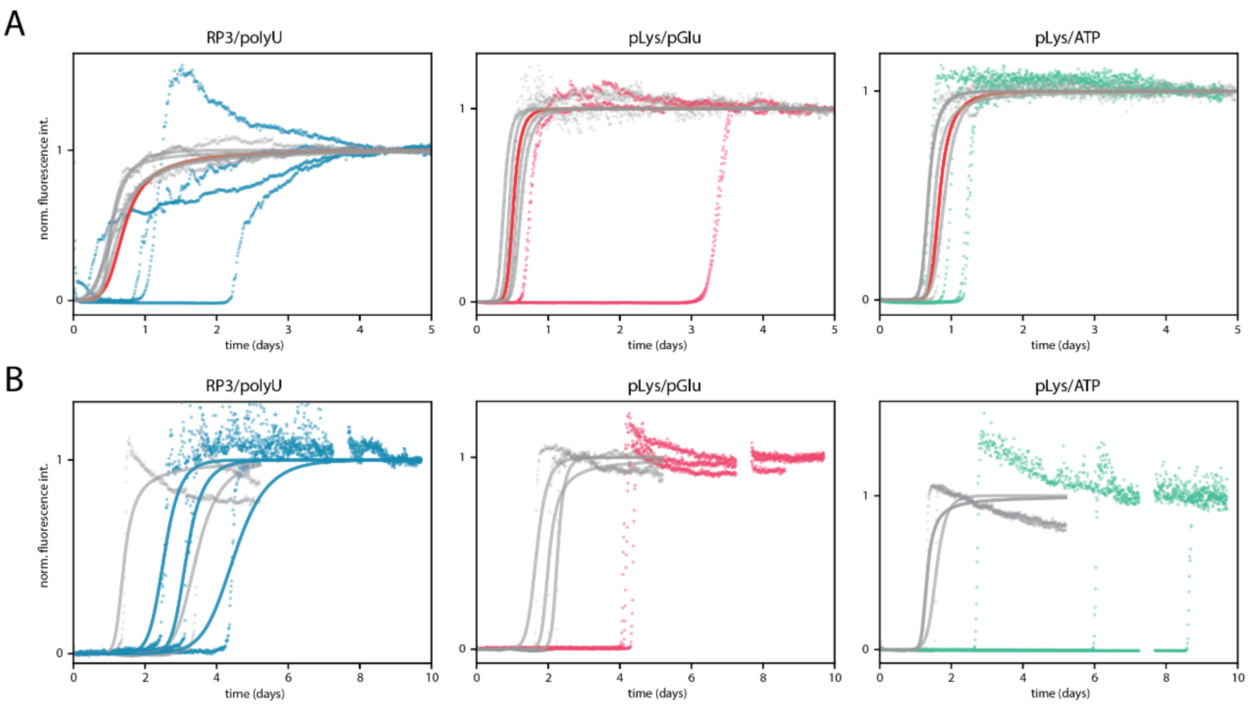

Fig. S6. Fitting of aggregation models to αSyn-108 and NACore aggregation. (**A**) Aggregation of αSyn-108 in the presence of different systems; supernatant traces with fitted curves are shown in grey (and average in red) and coacervate traces are shown in colour. (**B**) Aggregation of NACore in the presence of different systems; supernatant traces with fitted curves are shown in grey and coacervate traces are shown in colour. Proposed models for aggregation in the presence of coacervate systems can explain similar aggregation kinetics in the presence of droplets without partitioning, but fails to explain slower aggregation in the presence of droplets with low to moderate partitioning.

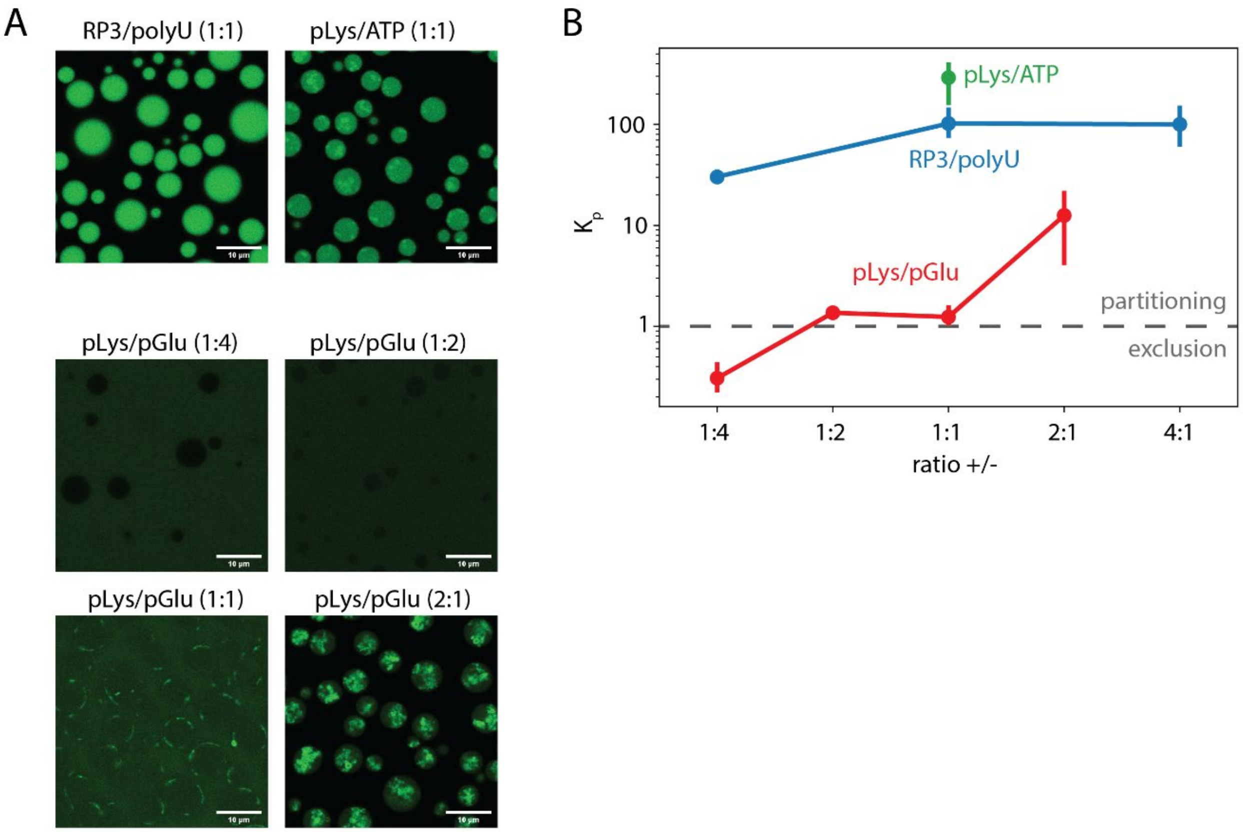

Fig. S7. Coacervates also interact differentially with insulin. (**A**) Confocal microscope images of coacervate systems with FAM- labelled insulin, colourised artificially. Ratio of positive to negative charge of the coacervate components is indicated in the brackets. (**B**) Partition coefficient of FAM-labelled insulin determined from microscopy experiments for different coacervate systems and different charge ratios of coacervate components.

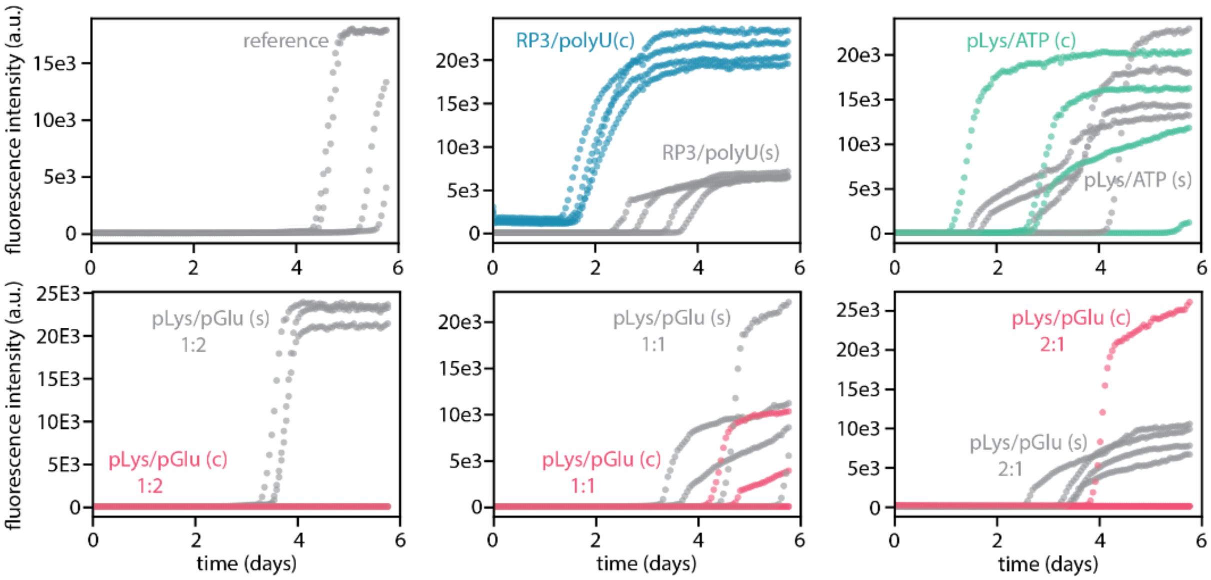

Fig. S8. Insulin aggregation is altered by coacervates. Aggregation traces (ThT fluorescence intensity) of insulin in buffer (reference, grey traces), in the presence of coacervates, or in the presence of coacervate supernatants (supernatant – s, coacervate – c).

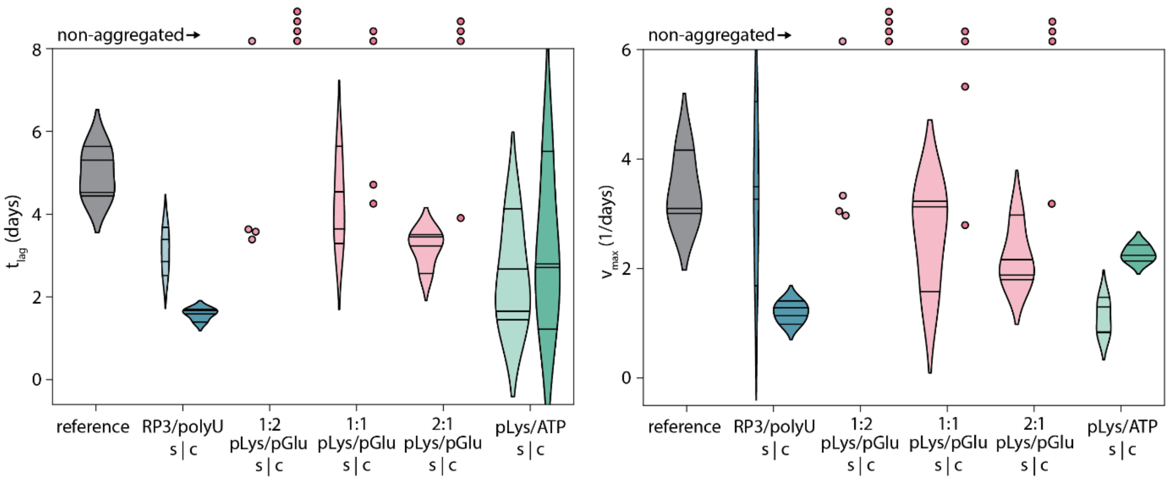

Fig. S9. Analysis of insulin aggregation kinetics. Distribution of the lag times (t_{lag}) and of the maximum aggregation rates (v_{max}) for insulin and all coacervate systems (supernatant – s, coacervate – c) and for the reference sample. Violin plots were prepared using Gaussian kernels with bandwidth determined automatically using Scott's method; density plots were cut at two bandwidth units past the extreme data points; violins are scaled to have the same area in supernatant-coacervate pairs.

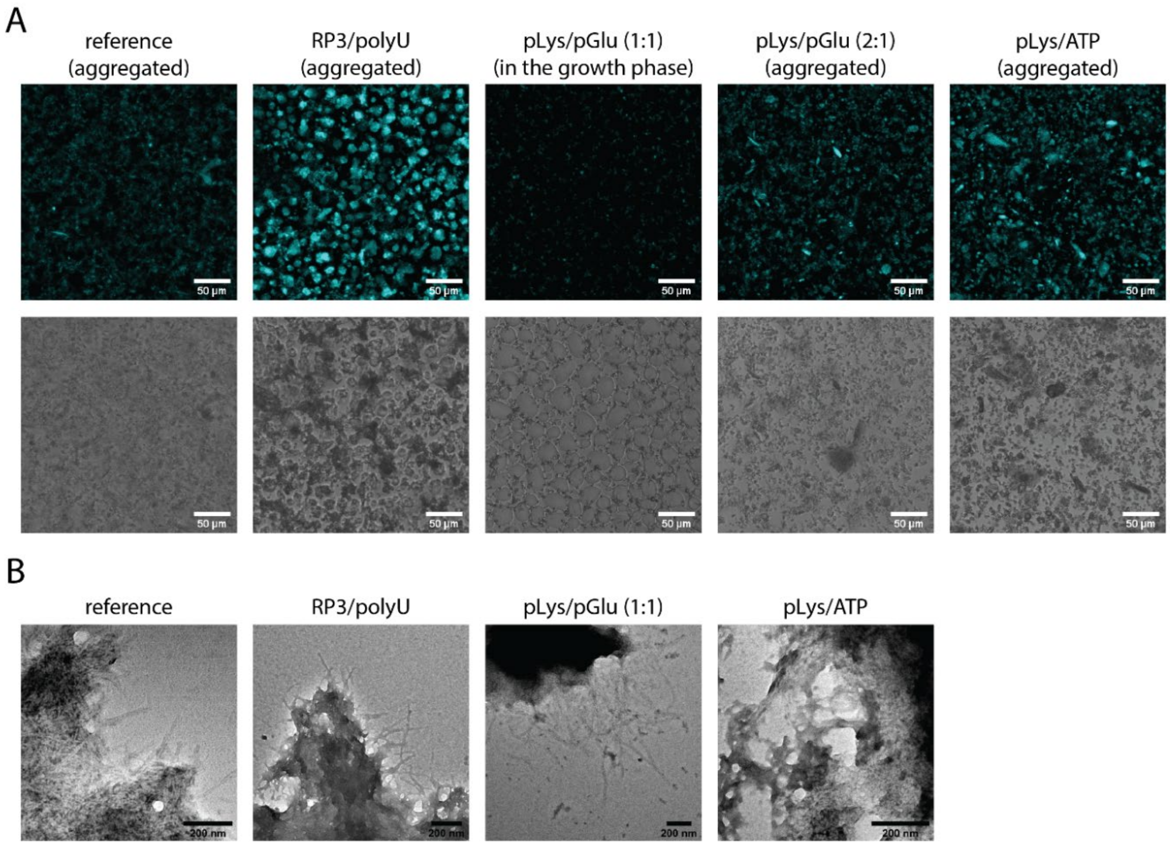

Fig. S10. Characteristics of insulin aggregates in the absence and presence of coacervates. (**A**) Confocal microscope fluorescence images and transmission images collected at the end of ThT aggregation assay (fig. S12). Apart from image for pLys/pGlu at 1:1 charge ratio, which was still in the growth phase, images show samples that reached aggregation plateau or were in the final stage of the growth phase. (**B**) TEM images of insulin aggregates formed in the presence of different coacervate systems. Insulin aggregates appear as fine fibrils.

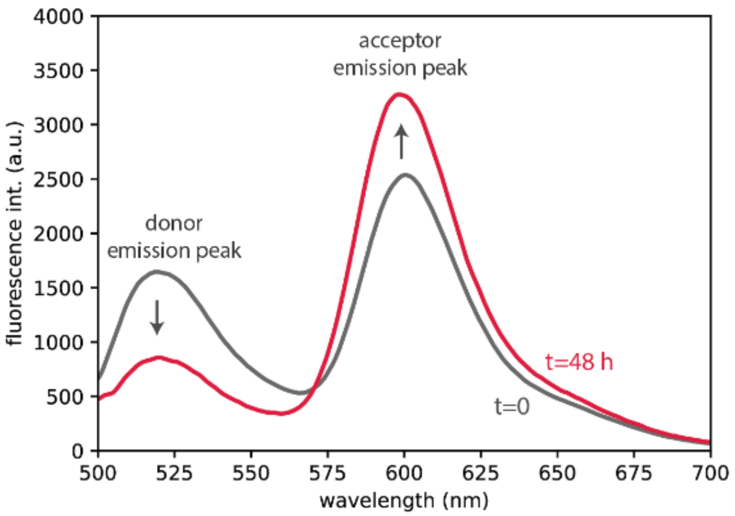

Fig. S11. An intramolecular FL-αSyn FRET probe reports on fibril formation. Fluorescence spectra of the FL-αSyn-based FRET probe in solution (in bulk), shortly after preparing the solution (*t*=0) and after 48 hours of incubation at 37 °C (*t*=48 h).

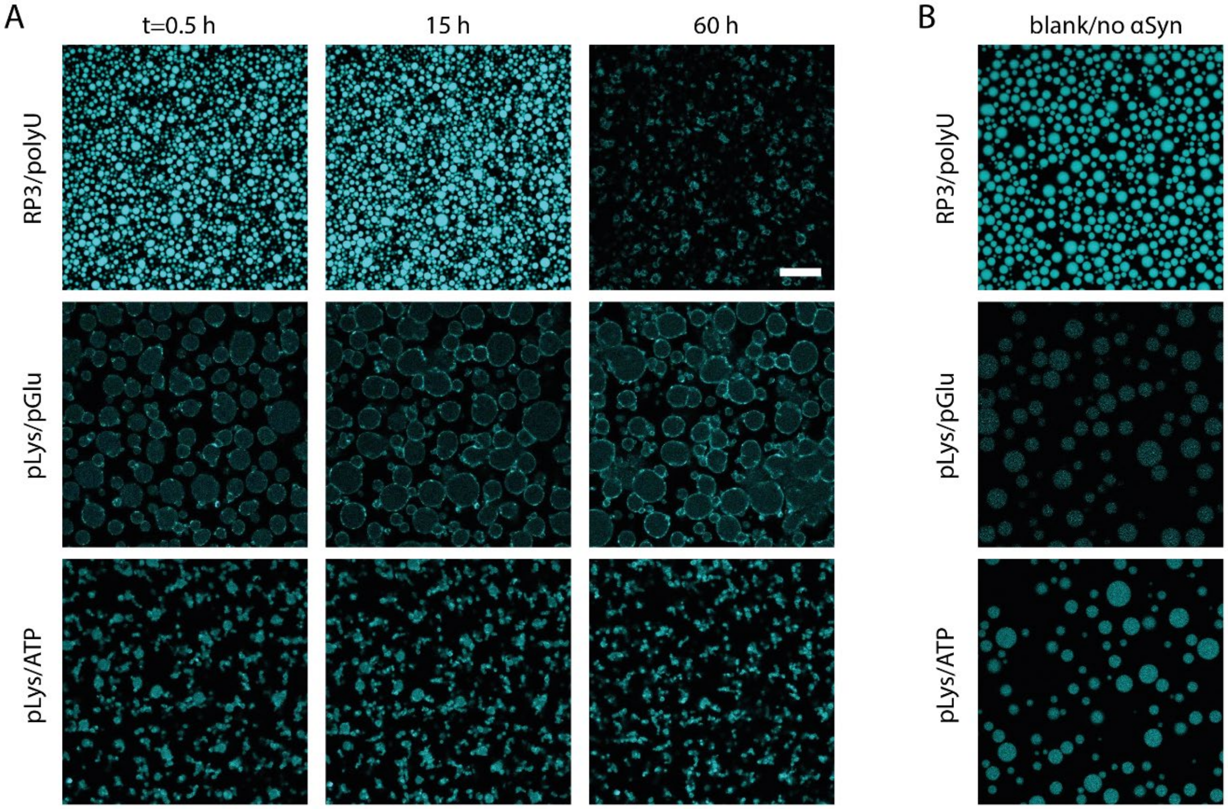

Fig. S12. Coacervates with aggregated FL-αSyn show different ThT signal from empty coacervate droplets. (**A**) ThT aggregation assay under confocal microscope of FL-αSyn in presence of different coacervate systems. (**B**) Partitioning of ThT into coacervate systems (without added FL-αSyn).

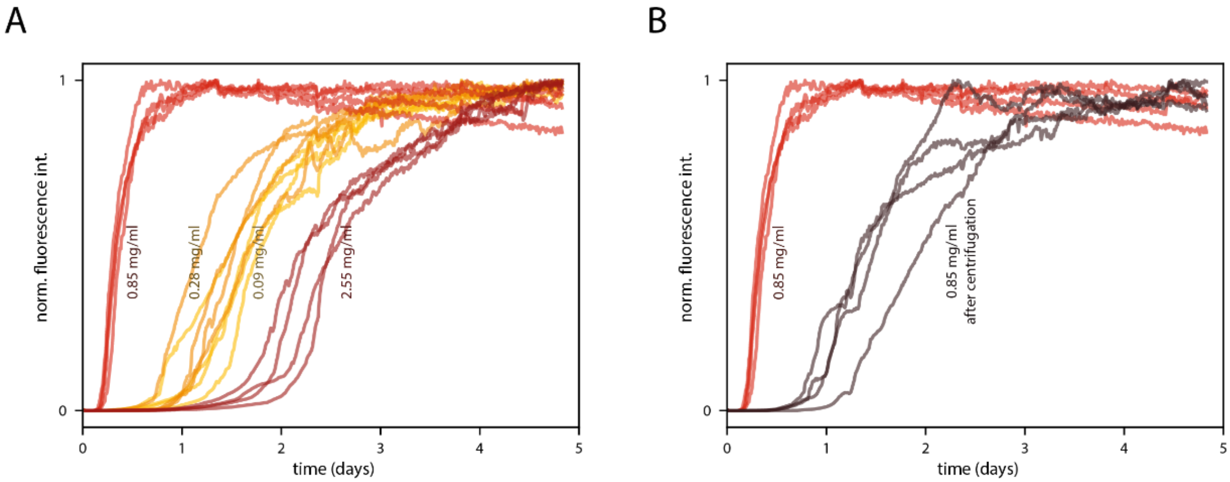

Fig. S13. Coacervate surface area affects FL-αSyn aggregation kinetics in the presence pLys/pGlu coacervates. (**A**) Aggregation traces of FL-αSyn in the presence of different amount of pLys/pGlu coacervates. (**B**) Aggregation traces of FL-αSyn in the presence of coacervates dispersed in solution and fused at the bottom of the plate after centrifugation.

Supplementary Model

Basic aggregation model

Typically for many amyloidogenic proteins, α-synuclein aggregation process may be considered an autocatalytic process. Our simple yet accurate model of α-synuclein aggregation is based on the secondary nucleation model proposed by Ferrone et al. (66) and involves 3 basic reactions: (i) primary nucleation of fibres from α-synuclein monomers, (ii) elongation of fibres by attaching monomers to one of the fibre ends, (iii) secondary nucleation catalysed by fibres:

$$r_{\text{primary nucleation}} = k_{\text{n}} \cdot [S]^n \tag{S1}$$

$$r_{\text{elongation}} = k_{+} \cdot [S] \cdot 2 \cdot [P] \tag{S2}$$

$$r_{\text{secondary nucleation}} = k_2 \cdot [S]^{n_2} \cdot [M] \tag{S3}$$

where: k_{n}, k_{+}, k_2 are the reaction rates of the corresponding reactions, n and n_2 are the nucleation numbers of primary and secondary nucleation (the lowest number of oligomers required to form a fibre nucleus), and $[S]$, $[P]$ and $[M]$ are the concentration of monomers, concentration of fibres (so $2 \cdot [P]$ reflects the number concentration of fibril ends) and concentration of monomeric units incorporated in fibres (proportional to fibre mass concentration and the surface available for secondary nucleation catalysis).

From this a set of differential equations describing concentration changes in the system can be derived:

$$\frac{d[S]}{dt} = -n \cdot k_{\text{n}} \cdot [S]^n - k_{+} \cdot [S] \cdot 2 \cdot [P] - n_2 \cdot k_2 \cdot [S]^{n_2} \cdot [M] \tag{S4}$$

$$\frac{d[P]}{dt} = k_{\text{n}} \cdot [S]^n + k_2 \cdot [S]^{n_2} \cdot [M] \tag{S5}$$

$$\frac{d[M]}{dt} = n \cdot k_{\text{n}} \cdot [S]^n + k_{+} \cdot [S] \cdot 2 \cdot [P] + n_2 \cdot k_2 \cdot [S]^{n_2} \cdot [M] \tag{S6}$$

Solving this set of equations provide a kinetic trace of the aggregation process. Fitting the solution to the experimentally measured concentration of one of the species provides information about the protein aggregation rates.

Aggregation in droplets model

In case of partitioning into the coacervate droplets, the concentrations of monomer in the diluted and in the condensed phase is determined by the partition coefficient:

$$K_{\mathrm{P}} = \frac{[S]_{\mathrm{cond}}}{[S]_{\mathrm{dil}}} \tag{S7}$$

where K_{P} is the partition coefficient and $[S]_{\mathrm{cond}}$ and $[S]_{\mathrm{dil}}$ are the concentrations of the monomer in the condensed and the diluted phase respectively. Taking into account the equation describing the mass balance of monomers in the system:

$$[S]_{\mathrm{tot}} = [S]_{\mathrm{dil}} \cdot \frac{R}{1+R} + [S]_{\mathrm{cond}} \cdot \frac{1}{1+R} \tag{S8}$$

where R is the ratio of diluted phase volume to the condensed phase volume, we can write equations describing the concentrations of the monomers in the diluted and in the condensed phase:

$$[S]_{\mathrm{dil}} = \frac{1+R}{R+K_{\mathrm{P}}} \cdot [S]_{\mathrm{tot}} = \xi \cdot [S]_{\mathrm{tot}} \tag{S9}$$

$$[S]_{\mathrm{cond}} = K_{\mathrm{P}} \cdot \frac{1+R}{R+K_{\mathrm{P}}} \cdot [S]_{\mathrm{tot}} = K_{\mathrm{P}} \cdot \xi \cdot [S]_{\mathrm{tot}} \tag{S10}$$

where $\xi = \frac{1+R}{R+K_{\mathrm{P}}}$. We assume the transport/partitioning process to be much faster than aggregation and to simplify the kinetic equations we assume further that the partitioning remains at equilibrium at every timepoint of the aggregation reaction. This leads to a set of differential equations describing aggregation process in the coacervate system with monomer partitioning:

$$\begin{aligned}\frac{d[S]_{\mathrm{tot}}}{dt} = &\left(\frac{R}{1+R}\right)\left[-nk_{\mathrm{n}}(\xi[S]_{\mathrm{tot}})^{n} - 2k_{+}\xi[S]_{\mathrm{tot}}[P]_{\mathrm{dil}} - n_2 k_2 (\xi[S]_{\mathrm{tot}})^{n_2}\,[M]_{\mathrm{dil}}\right] \\ &+ \left(\frac{1}{1+R}\right)\Big[-nk_{\mathrm{n_{cond}}}(K_{\mathrm{P}}\,\xi[S]_{\mathrm{tot}})^{n} - 2k_{+_{\mathrm{cond}}}K_{\mathrm{P}}\xi[S]_{\mathrm{tot}}[P]_{\mathrm{cond}} \\ &- n_2 k_{2_{\mathrm{cond}}}(K_{\mathrm{P}}\xi[S]_{\mathrm{tot}})^{n_2}\,[M]_{\mathrm{cond}}\Big]\end{aligned} \tag{S11}$$

$$\frac{d[P]_{\mathrm{dil}}}{dt} = k_{\mathrm{n}} \cdot (\xi[S]_{\mathrm{tot}})^{n} + k_2 \cdot (\xi[S]_{\mathrm{tot}})^{n_2}\,[M]_{\mathrm{dil}} \tag{S12}$$

$$\frac{d[M]_{\mathrm{dil}}}{dt} = nk_{\mathrm{n}}(\xi[S]_{\mathrm{tot}})^{n} + 2\,k_{+}\xi[S]_{\mathrm{tot}}\,[P]_{\mathrm{dil}} + n_2 k_2 (\xi[S]_{\mathrm{tot}})^{n_2}\,[M]_{\mathrm{dil}} \tag{S13}$$

$$\frac{d[P]_{\mathrm{cond}}}{dt} = k_{\mathrm{n_{cond}}}\,(K_{\mathrm{P}}\xi[S]_{\mathrm{tot}})^{n} + k_{2_{\mathrm{cond}}}(K_{\mathrm{P}}\,\xi[S]_{\mathrm{tot}})^{n_2}\,[M]_{\mathrm{cond}} \tag{S14}$$

$$\frac{d[M]_{\mathrm{cond}}}{dt} = nk_{\mathrm{n_{cond}}}(K_{\mathrm{P}}\xi[S]_{\mathrm{tot}})^{n} + 2k_{+_{\mathrm{cond}}}\,K_{\mathrm{P}}\xi[S]_{\mathrm{tot}}\,[P]_{\mathrm{cond}} + n_2k_{2_{\mathrm{cond}}}(K_{\mathrm{P}}\xi[S]_{\mathrm{tot}})^{n_2}\,[M]_{\mathrm{cond}} \tag{S15}$$

Again, similarly to the more simple case of aggregation in homogenous solution, solving the equations yields aggregation kinetic trace for both the diluted and the condensed phase. The proposed model is similar to the model previously described by Weber at al. (31), with the following main differences: we allow for different rate constants in the condensed and dilute phase, and we assume that the exchange of material between the droplet and the solution is infinitely fast.

Interface-aggregation model

Another model was developed for a case where aggregation-prone protein accumulates in the coacervate-diluted phase interface. Binding of the monomers to the coacervate interface can be described by equation:

$$K_{\mathrm{B}} = \frac{[S]_{\mathrm{int}}}{[S]_{\mathrm{dil}} \cdot [I]} \tag{S16}$$

where K_{B} is the binding constant, $[S]_{\mathrm{int}}$ is the concentration of interface-bound monomers and $[I]$ is the concentration of available binding sites ($[I] = [I]_{\mathrm{tot}} - [S]_{\mathrm{int}}$). Again, taking into account the mass balance equation for monomers, we can write equations describing the concentration of free and surface-bound monomers. Since the aggregation reaction occurs now only in the diluted phase (or in the interface, which is treated as a part of the diluted phase), we can omit the change of volume:

$$[S]_{\mathrm{dil}} = \frac{-1 - K_B[I]_{\mathrm{tot}} + [S]_{\mathrm{tot}}K_{\mathrm{B}} + \sqrt{\left(1 + K_{\mathrm{B}}([I]_{\mathrm{tot}} - [S]_{\mathrm{tot}})\right)^2 + 4K_{\mathrm{B}}[S]_{\mathrm{tot}}}}{2K_{\mathrm{B}}} \tag{S17}$$

$$[S]_{\mathrm{int}} = \frac{K_{\mathrm{B}}[I]_{\mathrm{tot}}[S]_{\mathrm{dil}}}{1 + K_{\mathrm{B}}[S]_{\mathrm{dil}}} \tag{S18}$$

We assume that the surface can act as a nucleation site, requiring one monomer from the surface and one monomer from the solution to react. If we further assume that the fibres formed at the

interface can grow by attaching monomers from the solution, they can participate in secondary nucleation and that they remain attached to the interface, we can write a set of differential equations for this system:

$$\frac{d[S]_{\mathrm{tot}}}{dt} = -nk_{\mathrm{n}}[S]_{\mathrm{dil}}{}^{n} - 2k_{+}[S]_{\mathrm{dil}}[P]_{\mathrm{dil}} - n_2k_2[S]_{\mathrm{dil}}{}^{n_2}[M]_{\mathrm{dil}} - 2k_{\mathrm{h}}[S]_{\mathrm{dil}}[S]_{\mathrm{int}} - k_{+_{int}}[S]_{\mathrm{dil}}[P]_{\mathrm{int}} - n_2k_{2_{int}}[S]_{\mathrm{dil}}{}^{n_2}[M]_{\mathrm{int}} \quad \text{(S19)}$$

$$\frac{d[P]_{\mathrm{dil}}}{dt} = k_{\mathrm{n}}[S]_{\mathrm{dil}}{}^{n} + k_2[S]_{\mathrm{dil}}{}^{n_2}[M]_{\mathrm{dil}} + k_{2_{int}}[S]_{\mathrm{dil}}{}^{n_2}[M]_{\mathrm{int}} \quad \text{(S20)}$$

$$\frac{d[M]_{\mathrm{dil}}}{dt} = nk_n[S]_{\mathrm{dil}}{}^{n} + 2k_{+}[S]_{\mathrm{dil}}[P]_{\mathrm{dil}} + n_2k_2[S]_{\mathrm{dil}}{}^{n_2}[M]_{\mathrm{dil}} + n_2k_{2_{int}}[S]_{\mathrm{dil}}{}^{n_2}[M]_{\mathrm{int}} \quad \text{(S21)}$$

$$\frac{d[P]_{\mathrm{int}}}{dt} = k_{\mathrm{h}}[S]_{\mathrm{dil}}[S]_{\mathrm{int}} \quad \text{(S22)}$$

$$\frac{d[M]_{\mathrm{int}}}{dt} = 2k_{\mathrm{h}}[S]_{\mathrm{dil}}[S]_{\mathrm{int}} + k_{+_{int}}[S]_{\mathrm{dil}}[P]_{\mathrm{int}} \quad \text{(S23)}$$

where k_{h} is the reaction rate constant of the interface-catalysed nucleation and, for clarity, $[S]_{\mathrm{dil}}$ and $[S]_{\mathrm{int}}$ symbols were used instead of full equations dependent on $[S]_{\mathrm{tot}}$.

The (local) concentration of monomers at the interface, $[S]_{\mathrm{int}}$, can be estimated from partitioning experiments (fig. 2) to be roughly 200 and 300 μM for the pLys/pGlu and pLys/ATP systems, respectively, which is low compared to the local concentration of pLys/pGlu or pLys/ATP inside the coacervates. Therefore, the use of a binding model that assumes single-layer adsorption seems justified.

REFERENCES AND NOTES

1. M. S. Hipp, P. Kasturi, F. U. Hartl, The proteostasis network and its decline in ageing. *Nat. Rev. Mol. Cell Biol.* **20**, 421–435 (2019).

2. G.-F. Chen, T.-H. Xu, Y. Yan, Y.-R. Zhou, Y. Jiang, K. Melcher, H. E. Xu, Amyloid beta: Structure, biology and structure-based therapeutic development. *Acta Pharmacol. Sin.* **38**, 1205–1235 (2017).

3. C. A. Brunello, M. Merezhko, R.-L. Uronen, H. J. Huttunen, Mechanisms of secretion and spreading of pathological tau protein. *Cell. Mol. Life Sci.* **77**, 1721–1744 (2020).

4. D. J. Irwin, V. M.-Y. Lee, J. Q. Trojanowski, Parkinson's disease dementia: Convergence of α-synuclein, tau and amyloid-β pathologies. *Nat. Rev. Neurosci.* **14**, 626–636 (2013).

5. S. Linse, Mechanism of amyloid protein aggregation and the role of inhibitors. *Pure Appl. Chem.* **91**, 211–229 (2019).

6. M. Törnquist, T. C. T. Michaels, K. Sanagavarapu, X. Yang, G. Meisl, S. I. A. Cohen, T. P. J. Knowles, S. Linse, Secondary nucleation in amyloid formation. *Chem. Commun.* **54**, 8667–8684 (2018).

7. T. C. T. Michaels, A. Šarić, J. Habchi, S. Chia, G. Meisl, M. Vendruscolo, C. M. Dobson, T. P. J. Knowles, Chemical kinetics for bridging molecular mechanisms and macroscopic measurements of amyloid fibril formation. *Annu. Rev. Phys. Chem.* **69**, 273–298 (2018).

8. D. Willbold, B. Strodel, G. F. Schröder, W. Hoyer, H. Heise, Amyloid-type protein aggregation and prion-like properties of amyloids. *Chem. Rev.* **121**, 8285–8307 (2021).

9. N. Laohakunakorn, L. Grasemann, B. Lavickova, G. Michielin, A. Shahein, Z. Swank, S. J. Maerkl, Bottom-up construction of complex biomolecular systems with cell-free synthetic biology. *Front. Bioeng. Biotechnol.* **8**, 213 (2020).

10. L. W. Simpson, T. A. Good, J. B. Leach, Protein folding and assembly in confined environments: Implications for protein aggregation in hydrogels and tissues. *Biotechnol. Adv.* **42**, 107573 (2020).

11. D. Ami, A. Natalello, M. Lotti, S. M. Doglia, Why and how protein aggregation has to be studied *in*

vivo. *Microb. Cell Fact.* **12**, 17 (2013).

12. D. M. Mitrea, R. W. Kriwacki, Phase separation in biology; Functional organization of a higher order Short linear motifs—The unexplored frontier of the eukaryotic proteome. *Cell Commun. Signal.* **14**, 1–20 (2016).

13. E. Gomes, J. Shorter, The molecular language of membraneless organelles. *J. Biol. Chem.* **294**, 7115–7127 (2019).

14. C. Greening, T. Lithgow, Formation and function of bacterial organelles. *Nat. Rev. Microbiol.* **18**, 677–689 (2020).

15. C. A. Azaldegui, A. G. Vecchiarelli, J. S. Biteen, The emergence of phase separation as an organizing principle in bacteria. *Biophys. J.* **120**, 1123–1138 (2021).

16. M. Feric, N. Vaidya, T. S. Harmon, D. M. Mitrea, L. Zhu, T. M. Richardson, R. W. Kriwacki, R. V. Pappu, C. P. Brangwynne, Coexisting liquid phases underlie nucleolar subcompartments. *Cell* **165**, 1686–1697 (2016).

17. T. E. Kaiser, R. V. Intine, M. Dundr, De novo formation of a subnuclear body. *Science* **322**, 1713–1717 (2008).

18. A. Molliex, J. Temirov, J. Lee, M. Coughlin, A. P. Kanagaraj, H. J. Kim, T. Mittag, J. P. Taylor, Phase separation by low complexity domains promotes stress granule assembly and drives pathological fibrillization. *Cell* **163**, 123–133 (2015).

19. A. A. Hyman, C. A. Weber, F. Jülicher, Liquid-liquid phase separation in biology. *Annu. Rev. Cell Dev. Biol.* **30**, 39–58 (2014).

20. T. J. Nott, T. D. Craggs, A. J. Baldwin, Membraneless organelles can melt nucleic acid duplexes and act as biomolecular filters. *Nat. Chem.* **8**, 569–575 (2016).

21. A. Patel, H. O. Lee, L. Jawerth, S. Maharana, M. Jahnel, M. Y. Hein, S. Stoynov, J. Mahamid, S. Saha, T. M. Franzmann, A. Pozniakovski, I. Poser, N. Maghelli, L. A. Royer, M. Weigert, E. W. Myers, S.

Grill, D. Drechsel, A. A. Hyman, S. Alberti, A liquid-to-solid phase transition of the ALS protein FUS accelerated by disease mutation. *Cell* **162**, 1066–1077 (2015).

22. S. Wegmann, B. Eftekharzadeh, K. Tepper, K. M. Zoltowska, R. E. Bennett, S. Dujardin, P. R. Laskowski, D. MacKenzie, T. Kamath, C. Commins, C. Vanderburg, A. D. Roe, Z. Fan, A. M. Molliex, A. Hernandez-Vega, D. Muller, A. A. Hyman, E. Mandelkow, J. P. Taylor, B. T. Hyman, Tau protein liquid–liquid phase separation can initiate tau aggregation. *EMBO J.* **37**, e98049 (2018).

23. J. Wen, L. Hong, G. Krainer, Q.-Q. Yao, T. P. J. Knowles, S. Wu, S. Perrett, Conformational expansion of Tau in condensates promotes irreversible aggregation. *J. Am. Chem. Soc.* **143**, 13056–13064 (2021).

24. S. Ray, N. Singh, R. Kumar, K. Patel, S. Pandey, D. Datta, J. Mahato, R. Panigrahi, A. Navalkar, S. Mehra, L. Gadhe, D. Chatterjee, A. S. Sawner, S. Maiti, S. Bhatia, J. A. Gerez, A. Chowdhury, A. Kumar, R. Padinhateeri, R. Riek, G. Krishnamoorthy, S. K. Maji, α-Synuclein aggregation nucleates through liquid–liquid phase separation. *Nat. Chem.* **12**, 705–716 (2020).

25. A. S. Sawner, S. Ray, P. Yadav, S. Mukherjee, R. Panigrahi, M. Poudyal, K. Patel, D. Ghosh, E. Kummerant, A. Kumar, R. Riek, S. K. Maji, Modulating α-Synuclein liquid-liquid phase separation. *Biochemistry* **60**, 3676–3696 (2021).

26. W. S. Woods, J. M. Boettcher, D. H. Zhou, K. D. Kloepper, K. L. Hartman, D. T. Ladror, Z. Qi, C. M. Rienstra, J. M. George, Conformation-specific binding of α-synuclein to novel protein partners detected by phage display and NMR spectroscopy. *J. Biol. Chem.* **282**, 34555–34567 (2007).

27. A. Esposito, C. P. Dohm, P. Kermer, M. Bähr, F. S. Wouters, α-Synuclein and its disease-related mutants interact differentially with the microtubule protein tau and associate with the actin cytoskeleton. *Neurobiol. Dis.* **26**, 521–531 (2007).

28. S. F. Banani, H. O. Lee, A. A. Hyman, M. K. Rosen, Biomolecular condensates: Organizers of cellular biochemistry. *Nat. Rev. Mol. Cell Biol.* **18**, 285–298 (2017).

29. K. K. Nakashima, M. A. Vibhute, E. Spruijt, Biomolecular chemistry in liquid phase separated compartments. *Front. Mol. Biosci.* **6**, 21 (2019).

30. C. Weber, T. Michaels, L. Mahadevan, Spatial control of irreversible protein aggregation. *Elife* **8** e42315 (2019).

31. A. M. Küffner, M. Linsenmeier, F. Grigolato, M. Prodan, R. Zuccarini, U. Capasso Palmiero, L. Faltova, P. Arosio, Sequestration within biomolecular condensates inhibits Aβ-42 amyloid formation. *Chem. Sci.* **12**, 4373–4382 (2021).

32. F. Grigolato, P. Arosio, The role of surfaces on amyloid formation. *Biophys. Chem.* **270**, 106533 (2021).

33. M. Grey, C. J. Dunning, R. Gaspar, C. Grey, P. Brundin, E. Sparr, S. Linse, Acceleration of α-synuclein aggregation by exosomes. *J. Biol. Chem.* **290**, 2969–2982 (2015).

34. C. Galvagnion, A. K. Buell, G. Meisl, T. C. T. Michaels, M. Vendruscolo, T. P. J. Knowles, C. M. Dobson, Lipid vesicles trigger α-synuclein aggregation by stimulating primary nucleation. *Nat. Chem. Biol.* **11**, 229–234 (2015).

35. M. Zhu, J. Li, A. L. Fink, The association of α-synuclein with membranes affects bilayer structure, stability, and fibril formation. *J. Biol. Chem.* **278**, 40186–40197 (2003).

36. S. Rocha, R. Kumar, I. Horvath, P. Wittung-Stafshede, Synaptic vesicle mimics affect the aggregation of wild-type and A53T α-synuclein variants differently albeit similar membrane affinity. *Protein Eng. Des. Sel.* **32**, 59–66 (2019).

37. A. S. Kurochka, D. A. Yushchenko, P. Bour, V. V. Shvadchak, Influence of lipid membranes on α-Synuclein aggregation. *ACS Chem. Nerosci.* **12**, 825–830 (2021).

38. W. M. Aumiller, C. D. Keating, Phosphorylation-mediated RNA/peptide complex coacervation as a model for intracellular liquid organelles. *Nat. Chem.* **8**, 129–137 (2016).

39. T. Ukmar-Godec, S. Hutten, M. P. Grieshop, N. Rezaei-Ghaleh, M. S. Cima-Omori, J. Biernat, E. Mandelkow, J. Söding, D. Dormann, M. Zweckstetter, Lysine/RNA-interactions drive and regulate biomolecular condensation. *Nat. Commun.* **10**, 2909 (2019).

40. I. Alshareedah, M. M. Moosa, M. Raju, D. A. Potoyan, P. R. Banerjee, Phase transition of RNA−protein complexes into ordered hollow condensates. *Proc. Natl. Acad. Sci. U.S.A.* **117**, 15650–15658 (2020).

41. S. L. Perry, L. Leon, K. Q. Hoffmann, M. J. Kade, D. Priftis, K. A. Black, D. Wong, R. A. Klein, C. F. Pierce III, K. O. Margossian, J. K. Whitmer, J. Qin, J. J. De Pablo, M. Tirrell, Chirality-selected phase behaviour in ionic polypeptide complexes. *Nat. Commun.* **6**, 6052 (2015).

42. S. Koga, D. S. Williams, A. W. Perriman, S. Mann, Peptide-nucleotide microdroplets as a step towards a membrane-free protocell model. *Nat. Chem.* **3**, 720–724 (2011).

43. K. K. Nakashima, J. F. Baaij, E. Spruijt, Reversible generation of coacervate droplets in an enzymatic network. *Soft Matter* **14**, 361–367 (2018).

44. A. K. Buell, C. Galvagnion, R. Gaspar, E. Sparr, M. Vendruscolo, T. P. J. Knowles, S. Linse, C. M. Dobson, Solution conditions determine the relative importance of nucleation and growth processes in α-synuclein aggregation. *Proc. Natl. Acad. Sci. U.S.A.* **111**, 7671–7676 (2014).

45. P. L. Onuchic, A. N. Milin, I. Alshareedah, A. A. Deniz, P. R. Banerjee, Divalent cations can control a switch-like behavior in heterotypic and homotypic RNA coacervates. *Sci. Rep.* **9**, 12161 (2019).

46. F. P. Cakmak, S. Choi, M. C. O. Meyer, P. C. Bevilacqua, C. D. Keating, Prebiotically-relevant low polyion multivalency can improve functionality of membraneless compartments. *Nat. Commun.* **11**, 5949 (2020).

47. K. K. Nakashima, M. H. I. van Haren, A. A. M. André, I. Robu, E. Spruijt, Active coacervate droplets are protocells that grow and resist Ostwald ripening. *Nat. Commun.* **12**, 3819 (2021).

48. T. Lu, E. Spruijt, Multiphase complex coacervate droplets. *J. Am. Chem. Soc.* **142**, 2905–2914 (2020).

49. P. M. McCall, S. Srivastava, S. L. Perry, D. R. Kovar, M. L. Gardel, M. V. Tirrell, Partitioning and enhanced self-assembly of actin in polypeptide coacervates. *Biophys. J.* **114**, 1636–1645 (2018).

50. T. Kaur, M. Raju, I. Alshareedah, R. B. Davis, D. A. Potoyan, P. R. Banerjee, Sequence-encoded and

composition-dependent protein-RNA interactions control multiphasic condensate morphologies. *Nat. Commun.* **12**, 872 (2021).

51. T. Lu, S. Liese, L. Schoenmakers, C. A. Weber, H. Suzuki, W. T. S. Huck, E. Spruijt, Endocytosis of coacervates into liposomes. *J. Am. Chem. Soc.* **144**, 13451–13455 (2022).

52. S. Elbaum-Garfinkle, Y. Kim, K. Szczepaniak, C. C.-H. Chen, C. R. Eckmann, S. Myong, C. P. Brangwynne, The disordered P granule protein LAF-1 drives phase separation into droplets with tunable viscosity and dynamics. *Proc. Natl. Acad. Sci. U.S.A.* **112**, 7189–7194 (2015).

53. A. Patel, L. Malinovska, S. Saha, J. Wang, S. Alberti, Y. Krishnan, A. A. Hyman, ATP as a biological hydrotrope. *Science* **356**, 753–756 (2017).

54. T. H. Kim, B. J. Payliss, M. L. Nosella, I. T. W. Lee, Y. Toyama, J. D. Forman-Kay, L. E. Kay, Interaction hot spots for phase separation revealed by NMR studies of a CAPRIN1 condensed phase. *Proc. Natl. Acad. Sci. U.S.A.* **118**, e2104897118 (2021).

55. N. A. Yewdall, A. A. M. André, M. H. I. van Haren, F. H. T. Nelissen, A. Jonker, E. Spruijt, ATP:Mg^{2+} shapes material properties of protein-RNA condensates and their partitioning of clients. *Biophys. J.* **121**, 3962–3974 (2022).

56. M. G. F. Last, S. Deshpande, C. Dekker, pH-controlled coacervate-membrane interactions within liposomes. *ACS Nano* **14**, 4487–4498 (2020).

57. N. Martin, M. Li, S. Mann, Selective uptake and refolding of globular proteins in coacervate microdroplets. *Langmuir* **32**, 5881–5889 (2016).

58. S. Lindhoud, L. Voorhaar, R. de Vries, R. Schweins, M. A. C. Stuart, W. Norde, Salt-induced disintegration of lysozyme-containing polyelectrolyte complex micelles. *Langmuir* **25**, 11425–11430 (2009).

59. W. C. Blocher McTigue, S. L. Perry, Protein encapsulation using complex coacervates: What nature has to teach us. *Small* **16**, 1907671 (2020).

60. N. A. Zervoudis, A. C. Obermeyer, The effects of protein charge patterning on complex coacervation. *Soft Matter* **17**, 6637–6645 (2021).

61. H. Naiki, K. Higuchi, M. Hosokawa, T. Takeda, Fluorometric determination of amyloid fibrils in vitro using the fluorescent dye, thioflavine T. *Anal. Biochem.* **177**, 244–249 (1989).

62. H. Levine III, Thioflavine T interaction with synthetic Alzheimer's disease β-amyloid peptides: Detection of amyloid aggregation in solution. *Protein Sci.* **2**, 404–410 (1993).

63. M. A. A. Fakhree, I. S. Nolten, C. Blum, M. M. A. E. Claessens, Different conformational subensembles of the intrinsically disordered protein α-Synuclein in cells. *J. Phys. Chem. Lett.* **9**, 1249–1253 (2018).

64. G. Veldhuis, I. Segers-Nolten, E. Ferlemann, V. Subramaniam, Single-molecule FRET reveals structural heterogeneity of SDS-bound α-synuclein. *Chembiochem* **10**, 436–439 (2009).

65. F. A. Ferrone, J. Hofrichter, W. A. Eaton, Kinetics of sickle hemoglobin polymerization. II. A double nucleation mechanism. *J. Mol. Biol.* **183**, 611–631 (1985).

66. G. Meisl, J. B. Kirkegaard, P. Arosio, T. C. T. Michaels, M. Vendruscolo, C. M. Dobson, S. Linse, T. P. J. Knowles, Molecular mechanisms of protein aggregation from global fitting of kinetic models. *Nat. Protoc.* **11**, 252–272 (2016).

67. E. Spruijt, J. Sprakel, M. A. Cohen Stuart, J. Van Der Gucht, Interfacial tension between a complex coacervate phase and its coexisting aqueous phase. *Soft Matter* **6**, 172–178 (2009).

68. M. Abbas, W. P. Lipiński, K. K. Nakashima, W. T. S. Huck, E. Spruijt, A short peptide synthon for liquid-liquid phase separation. *Nat. Chem.* **13**, 1046–1054 (2021).

69. W. Peeples, M. K. Rosen, Mechanistic dissection of increased enzymatic rate in a phase-separated compartment. *Nat. Chem. Biol.* **17**, 693–702 (2021).

70. S. Ray, D. Chatterjee, S. Mukherjee, K. Patel, J. Mahato, S. Kadam, R. Krishnan, A. S. Sawner, M. Poudyal, G. Krishnamoorthy, A. Chowdhury, R. Padinhateeri, S. K. Maj, Spatiotemporal solidification

of α-synuclein inside the liquid droplets. bioRxiv 2021.10.20.465113 [Preprint]. 21 October 2021. https://doi.org/10.1101/2021.10.20.465113.

71. V. N. Uversky, E. M. Cooper, K. S. Bower, J. Li, A. L. Fink, Accelerated α-synuclein fibrillation in crowded milieu. *FEBS Lett.* **515**, 99–103 (2002).

72. J. Goers, V. N. Uversky, A. L. Fink, Polycation-induced oligomerization and accelerated fibrillation of human α-synuclein in vitro. *Protein Sci.* **12**, 702–707 (2003).

73. T. Antony, W. Hoyer, D. Cherny, G. Heim, T. M. Jovin, V. Subramaniam, Cellular polyamines promote the aggregation of α-synuclein. *J. Biol. Chem.* **278**, 3235–3240 (2003).

74. M. E. Van Raaij, I. M. J. Segers-Nolten, V. Subramaniam, Quantitative morphological analysis reveals ultrastructural diversity of amyloid fibrils from α-synuclein mutants. *Biophys. J.* **91**, L96–L98 (2006).

75. T. Ishida, K. Kinoshita, PrDOS: Prediction of disordered protein regions from amino acid sequence. *Nucleic Acids Res.* **35**, W460–W464 (2007).

76. R. Dass, F. A. A. Mulder, J. T. Nielsen, ODiNPred: Comprehensive prediction of protein order and disorder. *Sci. Rep.* **10**, 14780 (2020).

77. B. Mészáros, G. Erdös, Z. Dosztányi, IUPred2A: Context-dependent prediction of protein disorder as a function of redox state and protein binding. *Nucleic Acids Res.* **46**, W329–W337 (2018).
